# Genetic Predisposition to Alzheimer’s Disease Is Associated with Enlargement of Perivascular Spaces in Centrum Semiovale Region

**DOI:** 10.3390/genes12060825

**Published:** 2021-05-27

**Authors:** Iacopo Ciampa, Grégory Operto, Carles Falcon, Carolina Minguillon, Manuel Castro de Moura, David Piñeyro, Manel Esteller, Jose Luis Molinuevo, Roderic Guigó, Arcadi Navarro, Juan Domingo Gispert, Natalia Vilor-Tejedor, for the ALFA Study

**Affiliations:** 1 Department of Radiology, Hospital Universitari Sagrat Cor, 08029 Barcelona, Spain; ciampa.iacopo@gmail.com; 2 Barcelonaβeta Brain Research Center (BBRC), Pasqual Maragall Foundation, 08005 Barcelona, Spain; goperto@barcelonabeta.org (G.O.); cfalcon@barcelonabeta.org (C.F.); cminguillon@barcelonabeta.org (C.M.); jlmolinuevo@barcelonabeta.org (J.L.M.); anavarro@barcelonabeta.org (A.N.); 3 IMIM (Hospital del Mar Medical Research Institute), 08003 Barcelona, Spain; 4 Centro de Investigación Biomédica en Red de Fragilidad y Envejecimiento Saludable (CIBERFES), 28029 Madrid, Spain; 5 Centro de Investigación Biomédica en Red Bioingeniería, Biomateriales y Nanomedicina, 28029 Madrid, Spain; 6 Josep Carreras Leukaemia Research Institute (IJC), 08916 Badalona, Barcelona, Spain; mcastro@carrerasresearch.org (M.C.d.M.); dpineyro@carrerasresearch.org (D.P.); mesteller@carrerasresearch.org (M.E.); 7 Centro de Investigación Biomedica en Red Cancer (CIBERONC), 28019 Madrid, Spain; 8 Institució Catalana de Recerca i Estudis Avançats (ICREA), 08010 Barcelona, Spain; 9 Physiological Sciences Department, School of Medicine and Health Sciences, University of Barcelona (UB), 08097 Barcelona, Spain; 10 Universitat Pompeu Fabra, 08005 Barcelona, Spain; roderic.guigo@crg.eu; 11 Centre for Genomic Regulation (CRG), Barcelona Institute of Science and Technology (BIST), 08003 Barcelona, Spain; 12 Department of Experimental and Health Sciences, Institute of Evolutionary Biology (CSIC-UPF), Universitat Pompeu Fabra, 08003 Barcelona, Spain; 13 Department of Clinical Genetics, Erasmus University Medical Center Rotterdam, 3015 GD Rotterdam, The Netherlands

**Keywords:** *APOE*-*ε4*, *BIN1*-rs744373, enlargement of perivascular spaces, neurogenetics, virchow robin spaces

## Abstract

This study investigated whether genetic factors involved in Alzheimer’s disease (AD) are associated with enlargement of Perivascular Spaces (ePVS) in the brain. A total of 680 participants with T2-weighted MRI scans and genetic information were acquired from the ALFA study. ePVS in the basal ganglia (BG) and the centrum semiovale (CS) were assessed based on a validated visual rating scale. We used univariate and multivariate logistic regression models to investigate associations between ePVS in BG and CS with *BIN1*-rs744373, as well as *APOE* genotypes. We found a significant association of the *BIN1*-rs744373 polymorphism in the CS subscale (*p* value = 0.019; OR = 2.564), suggesting that G allele carriers have an increased risk of ePVS in comparison with A allele carriers. In stratified analysis by *APOE*-*ε4* status (carriers vs. non-carriers), these results remained significant only for ε4 carriers (*p* value = 0.011; OR = 1.429). To our knowledge, the present study is the first suggesting that genetic predisposition for AD is associated with ePVS in CS. These findings provide evidence that underlying biological processes affecting AD may influence CS-ePVS.

## 1. Introduction

Perivascular spaces (PVS) are pial-lined and interstitial fluid-filled spaces in the brain surrounding the cerebral vessel walls that can be detectable in vivo by Magnetic Resonance Imaging (MRI) [1].

Enlargement of perivascular spaces (ePVS) in the brain is common but is generally overlooked and is of uncertain pathophysiology. Accumulated evidence suggests that ePVS correlates with aging [2], cognition [3], inflammatory processes [4], and cerebrovascular diseases [5], as well as with neurodegenerative pathologies [6,7]. Specifically, some previous studies have reported an association between ePVS and pathologic features of Alzheimer’s disease (AD) [8,9]. Other studies have reported that the frequency and severity of MRI-visible PVS are greater in AD than in cognitively unimpaired individuals [10,11,12]. However, the relationship between ePVS and AD is still poorly understood.

Identifying whether the genetic basis of AD influences ePVS in cognitively unimpaired individuals may provide additional insights into the neurobiological abnormalities that underlie AD.

The *Apolipoprotein E* (*Apo-E*) is a major cholesterol carrier that supports lipid transport. It also has an important role in Aβ metabolism, one of the pathological hallmarks of AD. It is well established that, even in asymptomatic AD stages, the *APOE-ε4* allele triggers Aβ accumulation not only in the brain parenchyma but also in the perivascular region, the latter leading to cerebral amyloid angiopathy (CAA), in which blood vessel function is disrupted [13]. In addition, amyloid-independent effects of *APOE* have been described on tau neurofibrillary degeneration, microglia and astrocyte responses, and blood-brain barrier disruption [14]. In particular, it has been recently shown that *APOE-ε4* can also increase blood-brain barrier permeability in the hippocampus and medial temporal lobe, contributing to cognitive decline independently of AD pathology [15]. However, controversial results have been found about its influence in ePVS [16,17].

Along with *APOE*, the *Bridging integrator 1* (*BIN1*) gene has been identified as an influential risk *locus* for AD [18,19]. *BIN1* is involved in the retrieval of synaptic vesicles, and ubiquitous isoforms of *BIN1* participate in inflammatory processes. Specifically, the *BIN1* rs744373 polymorphism has been reported as a modulator of tau clearance [20,21], which could provide a possible neural mechanism underlying the association between *BIN1* polymorphism and risk for AD. This genetic variant presents two possible alleles, A (major allele) and G (minor allele), the latter being associated with AD and thus considered the risk allele [22,23]. Although *BIN1* has been linked with lipid metabolism [24] and neuroinflammatory pathways [25], the exact pathogenic mechanisms of *BIN1* in the AD pathophysiological process remain to be determined, and no study to date has examined its involvement in ePVS.

**Figure 1:**
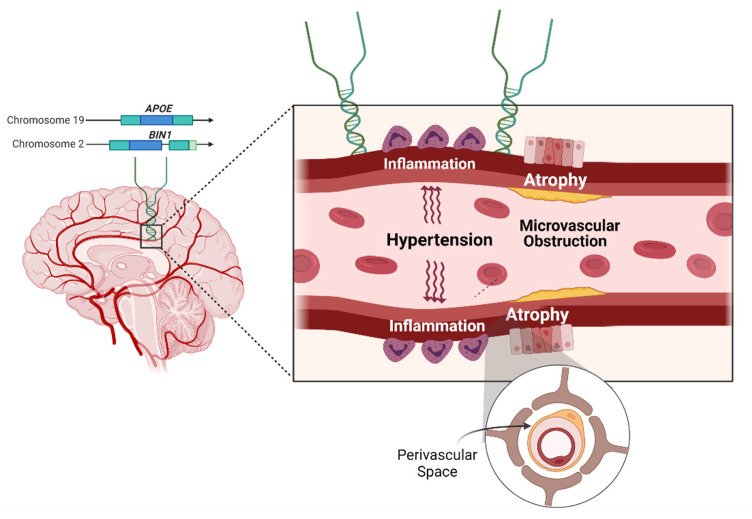
Schema of the study. Hypothesized etiologies for enlargement of perivascular spaces. Created with BioRender.com, accessed on 10–22 May 2021.

In this study, we aimed to investigate whether *APOE* and *BIN1* are associated with ePVS burden (Figure 1).

## 2. Material and Methods

### 2.1. Participants

Participants were drawn from the ALFA study (Alzheimer and Families) carried out in the Barcelonaβeta Brain Research Center [26]. The ALFA study is composed of 2743 cognitively unimpaired participants, mostly adult children of patients with AD, and aged between 45 and 75 years. A subset of 680 participants with available information on *BIN1*-rs744373 SNP and *APOE* genotypes, as well as having an MRI examination, were included in this study. The study sample is a large cohort of cognitively unimpaired individuals after an exhaustive neuropsychological and clinical screening procedure; therefore, results should not be confounded by comorbidities of dementia, being the individuals of the study are at a low mean of cardiovascular risk.

### 2.2. Standard Protocol Approvals, Registrations, and Patient Consents

The study was conducted in accordance with the directives of the Spanish Law 14/2007, of 3rd of July, on Biomedical Research (Ley 14/2007 de Investigación Biomédica). The ALFA study protocol was approved by the Independent Ethics Committee Parc de Salut Mar Barcelona and registered at Clinicaltrials.gov, accessed on 25 May 2021 (Identifier: NCT01835717). All participants accepted the study procedures by signing the study’s informed consent form that had also been approved by the same IRB.

### 2.3. Genotyping

Genome-wide genotyping was performed using the Illumina Infinium NeuroChip backbone [27], based on a genome-wide genotyping array (Infinium HumanCore-24 v1.0 and Infinium HumanCore-24 v1.2). PLINK was used for the quality control (QC) of genetic data [28]. We applied the following sample QC thresholds: sample missingness rates  > 2%, and heterozygosity less than 4 standard deviations. Additionally, we exclude individuals showing sex discordances and higher genetic relatedness (IBD > 0.185). Further details can be found in Reference [29]. The final genetic data set of the present study consisted of 680 participants of European ethnic origin with available information regarding *BIN1*-rs744373 polymorphism and *APOE* genotypes. Departures from Hardy-Weinberg equilibrium and allele frequencies were also inspected. The *APOE* allelic variants were obtained from allelic combinations of the rs429358 and rs7412 polymorphism [30]. According to the genotypes of these polymorphisms, subjects were classified depending on *APOE-ε4* status (non-carriers vs. carriers), the number of *ε4* alleles (non-carriers, one ε4 allele, or two *ε4* alleles), and *APOE* allelic variants (*ε3ε3*, *ε2ε3*, *ε3ε4*, and *ε4ε4*). Subjects were also classified depending on *BIN1*-rs744373 G allele status (non-carriers vs. carriers).

### 2.4. Image Acquisition and Rating of ePVS

Scans were obtained with a 3T scanner (Philips Ingenia CX, Eindhoven, Netherlands). The MRI protocol was identical for all participants and included high-resolution 3D T2-weighted structural images: Turbo Spin Echo, 256 × 256, 1 × 1 × 1 mm^3^ matrix, TR/TE: 2500/264 ms, flip angle = 90°. In addition, a 3D T1-weighted TFE sequence was acquired (voxel size 0.75 × 0.75 × 0.75 mm^3^, TR/TE: 9.90/4.6 ms, flip angle = 8°), as well as a 3D T2-FLAIR sequence (voxel size 1 × 1 × 1 mm^3^, TR/TE: 5000/312 ms). Scans were visually assessed for quality and incidental findings by a trained neuroradiologist.

ePVS were quantified independently in basal ganglia (BG) and centrum semiovale (CS) regions by a radiologist based on high-resolution T2-weighted images. The radiologist was blinded to other variables of the study. A visual rating scale used in previous publications [31,32,33,34] was used to code ePVS. Specifically, ePVS were assessed in the slice and hemisphere with the highest number, and rated as 0 (no PVS), 1 (mild; 1–10 PVS), 2 (moderate; 11–20 PVS), 3 (frequent; 21–40 PVS), or 4 (severe; >40 PVS).

Participants were dichotomized according to the severity of the ePVS rating of the BG and CS (degrees 0–2 were categorized as non-severe or 0; degrees 3–4 were categorized as severe or 1). The intra-rater agreement rate of the PVS scale was evaluated using a Kappa-Cohen agreement test on a random sample of 20% of the subjects in the dataset (κ = 0.77, *p* = 6.02 × 10^−8^ for BG subscale; and κ = 0.76, *p* = 8.2 × 10^−10^ for CS subscale).

### 2.5. Statistical Analysis

Differences in demographic variables were tested using the *χ*^2^ test and *F* test for gender, age, years of education, number of *APOE-ε4* alleles, and *BIN1*-rs744373 genotype.

The association between *APOE* genotypes and *BIN1* rs744373 polymorphism with the ePVS subscales and with the total scale were assessed by computing odds ratios (OR) using univariate and multivariate logistic regression models corrected by age, sex, and years of education. Dominant genetic models were assumed for *BIN1* rs744373. Briefly, in dominant models, homozygous of the major allele (i.e., AA genotype) were compared to heterozygous and homozygous of the minor allele (i.e., AG, GG genotypes).

For *APOE* genotype, we adjusted three models. In the first model, we compared *ε4* carriers vs. non-carriers (*APOE* status). In the second model, we compared individuals depending on the number of *ε4* alleles. Finally, in the third model, we compared *ε3ε3* individuals (reference category) vs. *ε2ε3*, *ε3ε4*, and *ε4ε4*. *APOE-ε2ε4* individuals were excluded in all analyses. Moreover, we additionally stratified the analysis of *BIN1* rs744373 polymorphism by *APOE-ε4* status, and we explored interaction effects between *BIN1* rs744373 and *APOE-ε4* status. To assess the association between demographic variables and ePVS, we additionally computed ORs using logistic regression models.

Statistical significance was set at False Discovery Rate (FDR) corrected *p* value < 0.05 (Benjamini-Hochberg procedure). All statistical analyses and data visualization were carried out using R version 3.4.4.

## 3. Results

### 3.1. Sample Descriptive

Table 1 and Table 2 show the characteristics of the sample. We divided the participants according to the severity of their ePVS rating in the BG and CS. We obtained two different categories for each region, with 550 non-severe and 130 severe BG ratings, and 227 non-severe and 453 severe CS ratings.

### 3.2. APOE and ePVS

The *APOE-ε4* allele was present in 280 individuals (41% of the sample). Of them, 239 (35% of the sample) were *APOE-ε4* heterozygous, and 41 (6% of the sample) *APOE-ε4* homozygous. In both subscales, the presence of *APOE*-*ε4* alleles was generally, more frequent in severe cases, while the *APOE-ε3ε3* genotype was, in general, more frequent in non-severe cases (Table 1 and Table 2).

We did not observe a significant association between *APOE* and ePVS. Specifically, significant differences between being an *ε4* carrier, and the ePVS subscales in both regions (BG *p* value = 0.379, CS *p* value = 0.445) were not found. In addition, we did not observe significant associations between the number of *ε4* alleles (0, 1, or 2) and the subscales (BG *p* value = 0.546 and 0.088, CS *p* value = 0.475 and 0.174). Finally, significant associations between *APOE* genotype and the subscales were not found either (Table 3).

### 3.3. BIN1-rs744373 and ePVS

**Figure 2:**
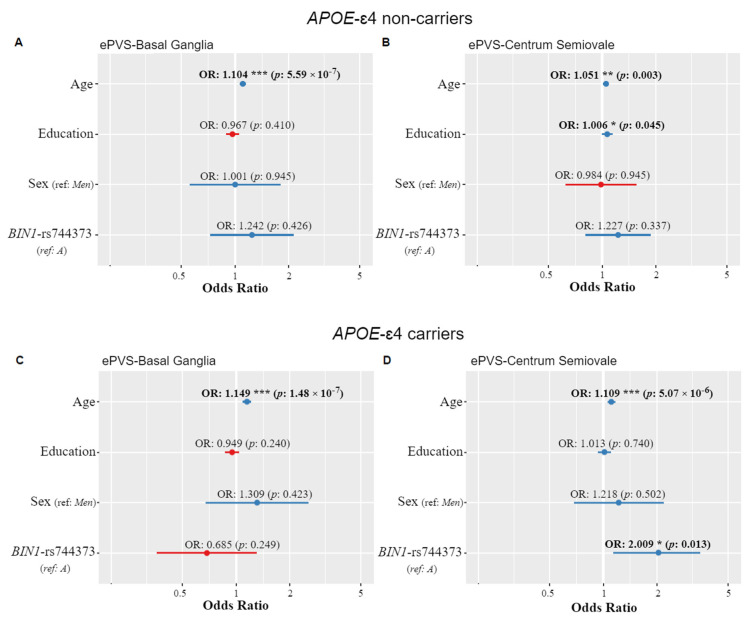
Associations (Odds Ratios) between enlargement of Perivascular Spaces in basal ganglia and centrum semiovale regions and *BIN1*-rs744373 polymorphism, stratified by *APOE* genotypes. Models were adjusted by age, sex, and years of education. *p*-values were corrected using the false discovery rate (FDR) method. *** *p*-value < 5 × 10^−5^; ** *p*-value < 5 × 10^−3^; * *p*-value < 5 × 10^−2^.

The *BIN1* rs744373-G allele was present in 345 individuals (51% of the sample). In the CS subscale, this allele was more frequent in the severe category (57%) than in the non-severe one (47%). We observed significant association of the *BIN1*-rs744373 SNP in the CS subscale (*p* value = 0.022; OR = 1.481), suggesting that G-allele carriers have an increased risk of PVS enlargement in comparison with A-allele carriers. These results remained significant in stratified analysis by *APOE-ε4* status, albeit only in *ε4* allele carriers (*p* value = 0.013; OR = 2.009) (Figure 2).

No significant associations were found in the BG region (Table 3), neither interactive significant associations between *BIN1*-rs744373 and *APOE* genotypes.

### 3.4. Age-Dependent Effects

We observed significant effects of age in both subscales (*p* value < 0.001), suggesting that older people are more likely to have ePVS in these regions irrespective of genetic predisposition to AD (Table 3). These results were independent of genetic associations (Figure 2). Years of education were significantly associated with CS-ePVS only in *APOE*-*ε4* non-carriers (OR = 1.01, *p* value = 0.045). No significant associations were found for sex.

## 4. Discussion

In this study, we investigated the association between ePVS and relevant genetic factors related to AD (i.e., the *APOE* genotype and *BIN1*-rs744373 polymorphism). We found significant associations between *BIN1*-rs744373 in the CS region, suggesting that G-allele carriers, which have OR = 1.7 of developing AD, have an increased risk of ePVS in comparison with A-allele carriers. These results were significant only in *APOE-ε4* carriers, suggesting that only those with a higher genetic predisposition to AD are associated with ePVS in CS. In addition, we did not find significant associations between *APOE* genotype or status and ePVS, which is in line with previous studies [11,35] and suggests that a multiple genetic predisposition to AD may affect ePVS.

To our knowledge, there were no previous studies that investigated the association of *BIN1* and ePVS. This gene is known for encoding a set of proteins generated by alternative RNA splicing with functions in membrane and actin dynamics, cell polarity, and stress signaling and has been identified as a relevant significant risk *locus* for late-onset AD [18,19]. Other studies have found that *BIN1* is involved in the neural degeneration of hippocampal, middle temporal, posterior cingulate, and precuneus regions, influencing the metabolism of glucose in the temporal lobe throughout the AD process [36]. Furthermore, other studies have found that the *BIN1 locus* is strongly associated with poorer memory performance without observing associations with brain MRI markers. These results led to the hypothesis that *BIN1* may exert its influence on the development of AD via mechanisms not visualized by structural MRIs, like amyloid-deposition or tau-pathology [37,38]. Indeed, other researchers found evidence that *BIN1* could contribute to the progression of AD-related tau pathology by altering tau clearance and promoting the release of tau-enriched extracellular vesicles by microglia via exosomes secretion [18] and by increasing aggregate internalization by endocytosis [37].

However, the characteristics of our study do not allow us to disentangle the molecular mechanisms associated with the observed effects of *APOE* and *BIN1* on ePVS. Unfortunately, biomarkers of AD pathology were not available in this sample to unravel whether the observed effects are mediated by amyloid and/or tau pathology. Given the main roles of *APOE* and *BIN1*, it could be speculated that, in *APOE-ε4* carriers, who are expected to harbor higher levels of amyloid pathology, the presence of the *BIN1*-rs744373 polymorphism can present with even further higher levels of amyloid and/or tau and lead to a disruption of the interstitial fluid dynamics in the brain. An alternative putative mechanism may involve the endosome-lysosome pathway resulting in an earlier and/or more severe AD-related neurodegeneration, which has also been associated with ePVS. However, the level of neurodegeneration in our participants is expected to be rather small, if any, given that they are cognitively unimpaired. Finally, since *APOE*, *BIN1*, and ePVS have all been reported to play an important role in the immune response of the brain, it could also be hypothesized that neuroinflammatory processes might be contributing to the effects observed in our work. Further studies, including biomarkers of core AD pathology and neuroinflammatory mechanisms, which are currently being collected in this sample, may help address these questions.

Another important aspect of our results is the dependence on ePVS topography. For instance, we found significant results in the CS region, but not in the BG. Interestingly, some previous studies reported that CS-ePVS appears to be associated with a clinical diagnosis of AD [12,39] (i.e., cerebral Aβ pathologies), whereas ePVS in the BG appears to be associated with subcortical vascular cognitive impairment [40,41]. However, literature on this is scarce, and some of these results were obtained from the analysis of heterogeneous populations (i.e., showing considerable differences correlating with the presence of cardiovascular risk factors). These differences make it difficult to extrapolate from the results, and this issue requires further research in homogenous populations.

A number of limitations in this study must be considered. Particularly challenging was the evaluation of MRIs with very small ePVS that can be seen as faint, indistinct, high signal structures, since those can cause a change from one category to another if considered. In addition, we should take into account that ePVS are a crude simplification of the complex anatomical brain patterns, and are not uniform across the lifespan. Moreover, the results should be interpreted considering the unavailability of a replication sample. Finally, the study sample belongs to a cognitively unimpaired population; thus, the identified associations cannot be interpreted to exert a causal relationship with the clinical presentation of AD.

However, the characteristics of the studied cohort, as well as the higher prevalence of the G allele of *BIN1*-rs744373 polymorphism and the *APOE-ε4* allele, allow us achieving an unprecedented statistical power in comparison with studies with similar number of individuals that are genetically closer to the general population [42].

In conclusion, our findings suggest that genetic risk factors for AD are associated with ePVS in CS. These results may provide evidence that the biological pathways affecting AD may influence ePVS in CS.

## Figures and Tables

**Table 1:** Characteristics of the study’s sample according to basal ganglia rating. Mean and SD are shown for continuous variables.

	Non-Severe BG Rating (*n* = 550)	Severe BG Rating (*n* = 130)	Total (*n* = 680)	*p* (χ^2^, F)
Age (m ± SD; years)	58.99 (±6.5)	64.02 (±5.81)	59.95 (±6.67)	0.120
Sex (female), *n* (%)	374 (68%)	86 (66%)	460 (67%)	0.763
Education (m ± SD; years)	13.58 (±3.42)	13 (±3.41)	13.47 (±3.42)	0.987
*APOE-ε4* carriers, *n* (%)	221 (40%)	59 (45%)	280 (41%)	0.324
Number of *APOE-ε4* alleles, *n* (%)	0:329 (60%); 1:192 (35%); 2:29 (5%)	0:71 (55%); 1:47 (36%); 2:12 (9%)	0:400 (59%); 1:239 (35%); 2:41 (6%)	0.195
*APOE-ε4* isoforms, *n* (%)	23:28 (5%); 33:297 (54%); 34:180 (33%); 44:29 (5%)	23:7 (5%); 33:60 (46%); 34:46 (35%); 44:12 (9%)	23:35 (5%); 33:357 (52%); 34:226 (33%); 44:41 (6%)	0.068
*BIN1-rs744373 G* *allele carriers n* (%)	280 (51%)	65 (50%)	345 (51%)	0.929

Legend: *n*, sample size; m, mean; SD, standard deviation; *p*, *p* value; BG, basal ganglia.

**Table 2:** Characteristics of the study’s sample according to Centrum Semiovale rating. Mean and SD are shown for continuous variables.

	Non-Severe CS Rating (*n* = 227)	Severe CS Rating (*n* = 453)	Total (*n* = 680)	*p* (χ^2^, F)
Age (m ± SD; years)	58.06 (±5.75)	60.9 (±6.9)	59.95 (±6.67)	0.002
Sex (female), *n* (%)	155 (68%)	305 (67%)	460 (67%)	0.87
Education (m ± SD; years)	13.21 (±3.47)	13.6 (±3.39)	13.47 (±3.42)	0.663
*APOE*-*ε4* carriers, *n* (%)	87 (38%)	193 (43%)	280 (41%)	0.323
Number of *APOE-ε4* alleles, *n* (%)	0:140 (62%); 1:77 (34%); 2:10 (4%)	0:260 (57%); 1:162 (36%); 2:31 (7%)	0:400 (59%); 1:239 (35%); 2:41 (6%)	0.348
*APOE* genotypes, *n* (%)	23:12 (5%); 33:126 (55%); 34:73 (32%); 44:10 (4%)	23:23 (5%); 33:231 (51%); 34:153 (34%); 44:31 (7%)	23:35 (5%); 33:357 (52%); 34:226 (33%); 44:41 (6%)	0.776
*BIN1-rs744373 G* *allele carriers n* (%)	130 (57%)	215 (47%)	345 (51%)	0.019

Legend: *n*, sample size; m, mean; SD, standard deviation; *p*, *p* value; CS, Centrum Semiovale.

**Table 3:** Associations between enlargement of Perivascular Spaces in basal ganglia and centrum semiovale. rs744373 polymorphisms and *APOE* genotype. Models were adjusted by age, sex, and education. ^₸^ False-discovery rate-corrected *p*-values.

	Basal Ganglia		Centrum Semiovale	
	OR (IC 95%)	*p*-Value ^₸^	OR (IC 95%)	*p*-Value ^₸^
Age (years)	1.121 [1.087;1.155]	<0.001	1.071 [1.043;1.099]	<0.001
Sex				
Male	Ref.	Ref.	Ref.	Ref.
Female	1.17 [0.721;1.626]	0.437	1.044 [0.743;1.474]	0.743
*APOE* genotypes				
*ε3ε3*	Ref.	Ref.	Ref.	Ref.
*ε2ε3*	1.256 [0.481;2.879]	0.620	1.039 [0.506;2.239]	0.918
*ε3ε4*	1.265 [0.822;1.937]	0.282	1.142 [0.803;1.631]	0.460
*ε4ε4*	2.057 [0.956;4.193]	0.064	1.672 [0.816;3.721]	0.165
				
*APOE-ε4*				
non-carriers	Ref.	Ref.	Ref.	Ref.
carriers	1.19 [0.839;1.818]	0.379	1.194 [0.862;1.658]	0.445
*APOE-ε4* genotypes				
0 alleles	Ref.	Ref.	Ref.	Ref.
1 allele	1.135 [0.750;1.706]	0.546	1.132 [0.806;1.596]	0.475
2 alleles	1.927 [0.902;3.893]	0.088	1.651 [0.809;3.663]	0.174
*BIN1*-rs744373 (dominant model)				
AA genotype	Ref.	Ref.	Ref.	Ref.
AG + GG genotype	1.037 [0.707;1.522]	0.848	1.481 [1.075;2.049]	0.022

Legend: Ref., Reference category; OR, Odds Ratio. Dominant models tested: *BIN1*-rs744373 GG group vs. *BIN1*-rs744373 GA and AA group.

## Data Availability

To protect participants’ privacy, individual level data cannot be made publicly available. Researchers who wish to use data from the ALFA study must obtain approval from the ALFA study Management Team (research@barcelonabeta.org).
